# Corrigendum

**DOI:** 10.1111/acel.13165

**Published:** 2020-05-27

In the original publication of the article “Implications of time‐series gene expression profiles of replicative senescence” by Y.‐M. Kim et al., the name of the first author, You‐Mie Kim, was misspelled as “You‐Mi Kim.”

The author would like to apologize for the inconvenience caused.
